# Anthropometric predictors of gestational hypertensive disorders in a remote aboriginal community: a nested case–control study

**DOI:** 10.1186/1756-0500-7-122

**Published:** 2014-03-05

**Authors:** Maryam Sina, Wendy Hoy, Zhiqiang Wang

**Affiliations:** 1 School of Medicine, University of Queensland, Postal address: Centre for Chronic diseases, Health Sciences Building, Royal Brisbane and Women Hospital, Herston, QLD 4029, Australia

**Keywords:** Gestational hypertensive disorders, BMI, Waist circumference, Aboriginal women

## Abstract

**Background:**

Australian Aboriginal women tend to have body shape and pregnancy risk profiles different from other Australian women. This study aims to examine the associations of anthropometric indices with gestational hypertensive disorders (GHD), and to determine the index that can best predict the risk of this condition occurring during pregnancy.

**Methods:**

This is a nested case–control study. Baseline body mass index (BMI), waist circumference (WC), hip circumference (HC), waist-to-hip ratio (WHR) and waist-to-height ratio (WHtR) were measured as part of a broader health screening program between 1992 and 1995 in a remote Aboriginal community. All subsequent pregnancies among the original participants were identified during 20 year follow-up period through hospital records (up to May 2012). Twenty eight women were diagnosed as having GHD, each of whom were individually matched by age at baseline with five women who were hospitalised for other pregnancy-related conditions and were free from GHD (n = 140). The associations of the baseline anthropometric measurements with GHD were assessed using conditional logistic regression.

**Results:**

The best predictor of GHD was WC (OR = 1.8; (95% CI, 1.1-2.9) for one standard deviation increase in WC), followed by BMI with the corresponding OR = 1.7 (95% CI, 1.1- 2.6). Other measurements, HC, WHR, and WHtR, were also positively associated with GHD, but those associations were not statistically significant.

**Conclusions:**

WC and BMI prior to pregnancy are anthropometric predictors of GHD in Aboriginal women, and WC is the best predictor. These findings imply the importance of early weight control in preventing GHD in Aboriginal women.

## Background

Body mass index (BMI) is a relatively reliable indicator of body fatness in clinical and epidemiological studies. However, different ethnic groups have different body fat distributions [1-3]. Aboriginal women tend to have larger waist circumferences (WC), waist-to-hip ratios (WHR) and waist-to-height ratios (WHtR) compared to non-Aboriginal Australians [4]. Other anthropometric measurements than BMI can also be important in epidemiologic studies undertaken in Aboriginal people [5-7] due to their unique body shapes, with long-legs in proportion to their body height and low sitting height [8].

Gestational hypertensive disorders (GHD) include gestational hypertension, pre-eclampsia and eclampsia. GHD is associated with an increased risk of chronic conditions such as type 2 diabetes mellitus [9]; it may also increase the risk of maternal and infant mortality [10,11]. These associations have been also reported in Australian Aborigines and Torres Strait Islanders (TSI) women [12]. A recent study undertaken in north Queensland showed that for every 1 cm increase in WC before pregnancy the risk of gestational hypertension increased by 4% [13]. That study also highlighted that for each one unit increase in BMI before pregnancy, the risk of gestational hypertension increased by 9%.

The impact of obesity on GHD during and before pregnancy has been reported by a number of studies [14,15]. However, the associations of different obesity-related measurements prior to pregnancy with GHD have not been systematically assessed. The objective of this study was to assess the associations of different anthropometric measurements with GHD in Aboriginal women.

## Methods

This is a nested case–control study. Cases and the matched-controls were derived from an existing cohort, established from members of one tribal group living remotely in the Northern Territory, Australia. Baseline anthropometric data were collected as part of a broader health screening program from 1992 to 1995. All participants were followed up to 31 May 2012, during which GHD were identified through hospital records. Two databases, the baseline screening of anthropometric measurements and a hospitalisation database of hypertensive outcomes were merged according to the patients’ hospital registration numbers. An informed written consent was obtained from all participants or guardians (for participants who were younger than 18 years old during the baseline measurements). The original baseline data collection was approved by the Aboriginal community and Menzies School of Health Research Ethics Committees. This project was approved by the University of Queensland Ethics Committee.

### Measurements

BMI was calculated as weight (kg) divided by height squared in meters. For participants older than 18 years at baseline, high BMI was defined as BMI ≥ 25 kg/m^2^. High BMI for participants aged less than 18 years at baseline were defined according to the international BMI cut-off points proposed by Cole et al. [16]. Since BMI changes substantially as a child gets older, and no BMI cut-off values have been developed specifically for Aboriginal children, we used the international BMI cut-off values, as used in previous studies of Aboriginal people [17]. The procedures for performing anthropometric measurements of weight, height, WC and hip circumference (HC) have been described in detail elsewhere [7]. Patients with baseline systolic blood pressure ≥ 140 mmHg and/or diastolic blood pressure ≥ 90 mmHg were considered as hypertensive. Pre-existing albuminuria was defined as a baseline urine albumin-to-creatinine ratio at or above the micro-albuminuria threshold of 3.4 mg/mmol [18].

### Pregnancy and GHD ascertainment

Women who became pregnant during the follow-up period were identified through their hospitalisation records according to the International Classification of Disease (ICD) 9 and ICD 10-AM codes, as shown in Table 1. GHD related ICD 10 codes were obtained according to ICD 10 codes used in Chen et al.’s study [19]. Women who were pregnant before the baseline measurements were excluded. A total of 173 women experienced pregnancy during the follow-up period. Of them, women with missing BMI data (n = 5) were excluded from this study. Of the remaining 168 women, 28 were identified with GHD, which was defined as having any of the following hypertensive conditions: gestational hypertension, preeclampsia, eclampsia, and pre-existing hypertension with superimposed pre-eclampsia-eclampsia. Participants with the missing other baseline measurements (i.e. WHR, HC) were excluded from the specific analysis (n = 61). Only the first identified records of GHD and the first identified records of other pregnancy-related conditions were extracted from the hospitalisation data.

**Table 1:** ICD 9 and ICD 10-AM codes for hospital diagnoses

**Condition of pregnancy**	**ICD-9 code**	**ICD-10-AM code**
Pregnancies without hypertension	630-633.9, 641–642, 643–650, 652–669, V22	Z34, O20-O29, O32-O48, O60-O75, O80-O85
Pregnancies with hypertension	642	O11, O13, O14, O15, O16

### Data analysis

Controls were individually matched to cases according to age at baseline. Continuous variables were tested for normality using skewness/Kurtosis tests. Most of those continuous variables were not significantly different from a normal distribution among cases; however this could be due to the small sample size. Therefore it is inconclusive whether those variables are normally distributed. To evaluate the associations of anthropometric measurements of BMI, WC, HC, WHR and WHtR as continuous variables with GHD, we used the conditional logistic regression method to calculate odds ratios (ORs) corresponding to one standard-deviation (SD) increase in each of those anthropometric measurements. The association between high BMI and GHD was also assessed using conditional logistic regression by calculating ORs and their 95% confidence intervals (CI). All models were adjusted for age at pregnancy, alcohol drinking and cigarette smoking. The anthropometric measurement with the highest OR value was considered as the best predictor of GHD. All analyses were conducted using Stata 12 [20].

## Results

### Baseline characteristics

Table 2 shows the mean values of baseline characteristics in cases and controls. The mean ages at baseline were 17.7 years (SD = 9.3) and 15.3 years (SD = 8.2) in cases and controls, respectively. The mean values of BMI were 22.4 kg/m^2^ (SD = 9.1) and 18.8 kg/m^2^ (SD = 5.7) in cases and controls, respectively. The mean value of WC was 88.5 cm (SD = 19.1) in cases and 78.6 cm (SD = 15.7) in controls.

**Table 2:** Patients’ baseline characteristics of the cases with gestational hypertension and the controls

**Characteristics**	**G-hypertension (case) 28**	**Other pregnant women (matched-control) 140**	***P-value***
	**Mean (SD)**	**Mean (SD)**	
Age at baseline	17.7 (9.3)	15.3 (8.2)	0.16
Age at pregnancy	24.6 (5.8)	22.4(6.2)	0.10
BMI (kg/m^2^)	22.4 (9.1)	18.8 (5.7)	0.007*
High BMI	11 (39.7%)	26 (18.5%)	0.016*
Waist circumference (cm)	88.5 (19.1)	78.6 (15.7)	0.013*
Hip circumference (cm)	98.2 (18.9)	89.2 (16.6)	0.029*
Waist-to-hip ratio^*^	0.92 (0.05)	0.89 (0.06)	0.10
Waist-to-height ratio	0.90 (0.06)	0.88 (0.07)	0.257
Systolic BP, mm Hg	109.4 (16.1)	104.7 (14.2)	0.09*
Diastolic BP, mm Hg	60.9 (14.1)	60.9 (11.7)	0.98
Pre-existing micro-albuminuria	10 (38.5%)	26 (19.1%)	0.030*
Gestational diabetes	10 (35.7%)	14 (10%)	<0.001**

*p<0.05; **p<0.001.

### Associations between anthropometric indices and GHD

The ORs are presented in Table 3. All five anthropometric indices were positively associated with GHD (Table 3). The association between WC and GHD was statistically significant, with crude OR = 1.8 (95% CI, 1.1-3.0). Furthermore, the association between BMI and GHD was also significant with crude OR = 1.7 (95% CI, 1.1-2.6). The associations of anthropometric indices with GHD in order of the magnitude of OR estimates were WC > BMI > HC ≈ WHR > WHtR (Table 3). Adjustment for age at pregnancy, alcohol drinking and cigarette smoking did not alter the order. The crude OR of high BMI for GHD was 2.8 (95% CI, 1.2-6.7) and adjusted OR was 2.7 (95% CI, 1.0-7.3).

**Table 3:** Associations of different anthropometric measurements with gestational hypertensive disorders

	***Crude***			Adjusted**		
	OR*	**95% CI**	**P-value**	OR*	**95% CI**	**P-value**
BMI	1.69	1.11, 2.57	0.014***	1.68	1.10, 2.58	0.016***
Waist circumference	1.82	1.12, 2.95	0.014***	1.78	1.10, 2.89	0.019***
Hip circumference	1.60	0.99, 2.57	0.053	1.53	0.96, 2.52	0.068
Waist-to-hip ratio	1.57	0.97, 10.4	0.056	1.65	0.80, 3.39	0.174
Waist-to-height ratio	1.43	0.84, 2.46	0.186	1.44	0.83, 2.51	0.192

*Odds ratios corresponding to one standard-deviation increase in BMI, waist circumference, and waist-to-hip ratio. Sample size varies by the associations, because of the missing information of anthropometric indices.

**Adjusting for age at pregnancy, alcohol drinking and cigarette smoking.

***p<0.05.

## Discussion

Our findings suggest that WC, prior to pregnancy, is the best anthropometric predictor of GHD in Aboriginal women. All five indices are positively associated with GHD, and can be used to predict GHD several years prior to pregnancy. However, only the associations of BMI and WC with GHD are statistically significant, before and after adjusting for confounding factors. Moreover, women with high BMI before pregnancy have over twice the odds of experiencing GHD during pregnancy.

Although there are a number of studies have reported the impact of BMI, both before and during pregnancy, on GHD [14,15,21], the associations of anthropometric measurements, other than BMI, several years before pregnancy with GHD have not been well reported. Aboriginal Australians are more likely to experience pregnancy related complications [22,23]; and they also tend to be obese in young adulthood [24]. Therefore, understanding the associations of GHD with different anthropometric measurements, prior to pregnancy, is potentially a useful method to identify high risk individuals [22]. This study is the first that assessed the associations of five anthropometric measurements prior to pregnancy with GHD, in Aboriginal women. Moreover, the data used in this study were obtained from a cohort with up to 20 years of follow-up, and the anthropometric measurements were undertaken several years (mean = 7 years) prior to pregnancy. Therefore our findings could be valid evidence in terms of the impacts of WC and BMI several years prior to pregnancy on GHD.

Campbell *et al.* have also assessed the impact of WC and BMI before pregnancy on the development of gestational hypertension [13]. Their results, similar to ours, showed significant associations of WC and BMI with GHD. The current study is also comparable with a study undertaken by Lake *et al.* In their study, the impact of BMI before pregnancy on development of GHD was determined among women who became pregnant by the age of 33 [25]. In that study, BMI measurements were carried out twice, at seven and 23 years of age. Their results showed a positive association between obesity at the age of seven and GHD. Lake *et al*. also reported a positive association between obesity at the age of 23, among women who experienced pregnancy by the age of 33, and GHD, independent of their BMI at the age of seven [25].

Ijuin *et al.* have investigated the association between upper/lower-half-body ratio (body-fat amount ratio) and pre-eclampsia. Their findings could be comparable with ours in regard to association between WHR and GHD. According to their study, upper/lower-half-body ratio is associated with pre-eclampsia [26]. Both studies showed a positive association and a very large unadjusted point estimate. However, our findings did not show a significant association between WHR and GHD after adjusting for confounding factors. Furthermore, their results showed a weak association between BMI and pre-eclampsia, whereas a number of studies including ours showed a strong association even after adjusting for other risk factors.

In this study all participants were from a relatively homogenous Aboriginal community, which simplified the comparison between cases and controls, although there are no standardised BMI or WC cut-off points for this population.

One of the limitations of this study was its small sample size. In addition, because the number of missing data for some of the measurements (e.g. WHR, WHtR) was higher than the number of missing BMI data, the related assessments had a smaller sample-size than the original study groups, which reduced the related statistical power. In addition, because women with GHD in this study were identified through hospital records, minor GHD cases might had not been hospitalised, and misclassified as controls, which could have diluted the true associations between the anthropometric indices and GHD. There is also a possibility of human error in performing the anthropometric measurements. Since this study was conducted in one remote community, the generalisability of our findings to other Aboriginal communities remains to be verified.

## Conclusions

There are positive associations between GHD in Australian Aboriginal women and their BMI and WC status several years prior to pregnancy. The findings of this study could have multiple clinical and public health implications. It is useful for clinical practitioners and the general public to understand the long term risk of high BMI and WC on GHD. In order to reduce the risk of GHD developing in Aboriginal women, it is important to prevent high BMI in children and young adults.

## Competing interests

The authors declare they have no competing interests.

## Authors’ contributions

The contributions of each author in this work are as follows: MS drafted the manuscript and performed data analysis. MS, WH, ZW contributed to study design. WH and ZW obtained the data and provided guidance for data analysis and interpretation. All authors critically reviewed, revised and approved the final draft of the manuscript.
